# Dr. Louis A. Weigel

**Published:** 1904-11

**Authors:** 


### Dr. Louis A. Weigel.

ONE of the saddest incidents of prolonged experimentation with Röntgen rays is that which has resulted in depriving Dr. Weigel of his right hand and part of his left, on account of *x*-ray burns.

Dr. Weigel, who is a prominent orthopedic surgeon residing at Rochester, and a former president of the American Orthopedic Association, has been an enthusiastic amateur photographer, employing it chiefly in illustrating or diagnosticating injuries and deformities in the young. It is stated that frequently the skin on his hands became irritated or abraded with the developing chemicals and that, as a consequence, they were more susceptible to the influence of the *x*-rays. At all events, near the beginning of the present year he noticed certain ill effects pertaining to the frequent use of the rays, the manifestation beginning in one of his hands.

A few weeks ago he came to Buffalo and consulted Dr. Wende, remaining some time under his care. Some improvement followed which gave encouragement to Dr. Weigel and his friends, but again there came developments which strongly suggested the appeal to surgery. Accordingly, Dr. Weigel returned to Rochester, where at the city hospital, October 11, 1904, a double synchronous amputation was made of the right hand and three fingers of the left, by Dr. Roswell Park, of Buffalo, and Dr. William B. Coley, of New York. Dr. Taylor, of New York, and Dr. Wende, of Buffalo, as well as some others, were present.

We understand that the distinguished patient has progressed favorably since the operation, with fair prospects of ultimate recovery. Dr. Weigel has the sympathy of a large professional circle in this distressing calamity, and the hope is widely expressed that his recovery may now be complete.

